# Construction and analysis of a survival-associated competing endogenous RNA network in breast cancer

**DOI:** 10.3389/fsurg.2022.1021195

**Published:** 2023-01-06

**Authors:** Gang Chen, Yalun Li, Jianqiao Cao, Yuanping Dai, Yizi Cong, Guangdong Qiao

**Affiliations:** ^1^ Department of Breast Surgery, The Affiliated Yantai Yuhuangding Hospital of Qingdao University, Yantai, China; ^2^ Department of Medical Genetics, Liuzhou Maternal and Child Health Hospital, Liuzhou, China

**Keywords:** bioinformatics, breast cancer, prognosis biomarker, GEO, ceRNAs

## Abstract

**Background:**

Recently, increasing studies have shown that non-coding RNAs are closely associated with the progression and metastasis of cancer by participating in competing endogenous RNA (ceRNA) networks. However, the role of survival-associated ceRNAs in breast cancer (BC) remains unknown.

**Methods:**

The Gene Expression Omnibus database and The Cancer Genome Atlas BRCA_dataset were used to identify differentially expressed RNAs. Furthermore, circRNA-miRNA interactions were predicted based on CircInteractome, while miRNA-mRNA interactions were predicted based on TargetScan, miRDB, and miRTarBase. The survival-associated ceRNA networks were constructed based on the predicted circRNA-miRNA and miRNA-mRNA pairs. Finally, the mechanism of miRNA-mRNA pairs was determined. Gene Ontology (GO) and Kyoto Encyclopedia of Genes and Genomes (KEGG) analyses of survival-related mRNAs were performed using the hypergeometric distribution formula in R software.The prognosis of hub genes was confirmed using gene set enrichment analysis.

**Results:**

Based on the DE-circRNAs of the top 10 initial candidates, 162 DE-miRNAsand 34 DE-miRNAs associated with significant overall survival were obtained. The miRNA target genes were then identified using online tools and verified using the Cancer Genome Atlas (TCGA) database. Overall, 46 survival-associated DE-mRNAs were obtained. The results of GO and KEGG pathway enrichment analyses implied that up-regulated survival-related DE-mRNAs were mostly enriched in the “regulation of cell cycle” and “chromatin” pathways, while down-regulated survival-related DE-mRNAs were mostly enriched in “negative regulation of neurotrophin TRK receptor signaling” and “interleukin-6 receptor complex” pathways. Finally, the survival-associated circRNA-miRNA-mRNA ceRNA network was constructed using 34 miRNAs, 46 mRNAs, and 10 circRNAs. Based on the PPI network, two ceRNA axes were identified. These ceRNA axescould be considered biomarkers for BC.GSEA results revealed that the hub genes were correlated with “VANTVEER_BREAST_CANCER_POOR_PROGNOSIS”, and the hub genes were verified using BC patients' tissues.

**Conclusions:**

In this study, we constructed a circRNA-mediated ceRNA network related to BC. This network provides new insight into discovering potential biomarkers for diagnosing and treating BC.

## Introduction

Breast cancer (BC) is a common malignancy among women worldwide (1). Female BC mortality peaked in 1989 and has since fallen by 42% due to earlier diagnosis and treatment advancements (2), with 5-year survival rates for all stages reaching up to 90% (2). Despite high survival rates in women, there is still a lack of suitable molecular biomarkers for early diagnosis and targets for precision therapy.

Circular RNAs (circRNAs) are novel non-coding RNAs produced by precursor mRNA back-splicing or skipping events. They lack 5′ caps and 3′ poly(A) tails (3, 4) and are widely expressed across species. Owing to their high abundance, evolutionary conservation, and stability, circRNAs could be biomarkers for clinical diseases (3, 5). Several studies have shown that circRNAs correlate with carcinogenesis and patient progression (6–8); for example, circHMGCS1 could promote hepatoblastoma cell growth (9), and circANKS1B induces BC metastasis regulated by ESRP1 (10). Although few studies have reported differential expression of circRNAs, the potential biological function and underlying mechanisms of circRNAs remain unclear.

MicroRNAs (miRNAs) are highly conserved small non-coding RNAs regulate gene expression through posttranslational inhibition or target mRNA degradation (3). miRNAs could act as tumor inhibitors in various cancers, including BC (11, 12). However, the upstream mechanisms of miRNAs remain unclear. The competing endogenous RNA (ceRNA) theory, in which non-coding RNAs regulate mRNA expression by competitively binding to shared microRNA response elements (MREs), has been hypothesized (13). According to some studies, circRNAs are a new member of the ceRNA family with a key role in cancer gene expression and regulation (14, 15).

**Figure 1:**
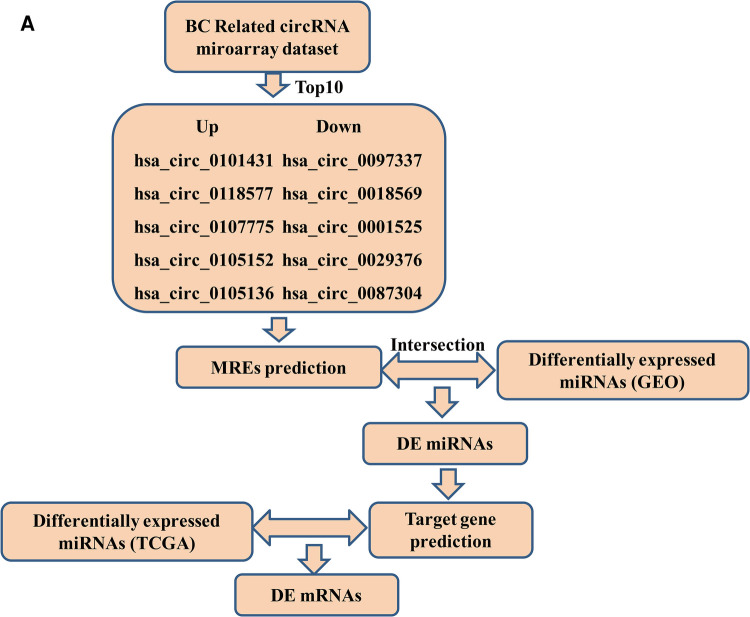
Flow chart of the ceRNA network analysis.

Here, we constructed a ceRNA network associated with survival in differentially expressed (DE) genes to demonstrate the molecular mechanisms underlying BC. CircRNA (GSE101123, GSE101124) and miRNA (GSE97811) expression profiles were obtained from the Gene Expression Omnibus (GEO) database. The top ten DE circRNAs were then selected as initial candidates based on their *p*-value. The prognosis-related circRNA–miRNA–mRNA network was then constructed based on circRNA-miRNA interactions and miRNA-mRNA interactionspredicted using online tools. Furthermore, Gene Ontology (GO) and Kyoto Encyclopedia of Genes and Genomes (KEGG) pathway analyses were performed to explore the mechanism of DE mRNAs. The hub genes of mRNAs were filtered out using the Cytoscape cytoHubba plugin. Finally, an integrated circRNA-mRNA network was successfully constructed. The ceRNA network provides an exact and reliable result for further studies. A concise workflow outline of the procedure is depicted in Figure 1.

## Materials and methods

### Microarray data information and identification of the De circRNAs, miRNAs, and mRNAs

The expression data of circRNAs, miRNAs, and mRNAs were obtained from the GEO database (http://www.ncbi.nlm.nih.gov/geo/), an international public and research repository platform. GSE101123 and GSE101124, based on the GPL19978 platform,are circRNA databases of BC, and both contain data from eight cancer tissue specimens and three normal tissue specimens. GSE97811 is a miRNA database that includes 45 cancer and 16 normal tissues based on the GPL21263 platform. The Cancer Genome Atlas (TCGA) TCGA_BRCA mRNA dataset,which contained 1,102 cancer tissues and 113 normal tissues, was downloaded using theTCGA-Assembler package (Version 2.0) based on the gene.normalized_RNAseqassay platform. Table 1 provides basic information on these gene expression profiles.

**Table 1:** Basic information from the GEO microarray and TCGA RNA-seq datasets.

Data source	Platform	Tissue	Sample size (Tumor/Normal)	Author	Year	Region	RNA type
GSE101123	GPL19978	Breast	8/3	Jianzhen Xu	2018	China	CircRNA
GSE101124	GPL19978	Breast	8/3	Jianzhen Xu	2018	China	CircRNA
GSE97811	GPL21263	Breast	45/16	AI Mitsuhashi	2017	Japan	miRNA
TCGA_BRCA	gene.normalized_RNAseq	Breast	1102/113			USA	mRNA

The gene normalized signal intensity contained in the series matrix file and GPL probe information text was retrieved from each gene chip based on the GEO database. The circle database Hg19 was obtained from Circbase (http://www.circbase.org/) database. The NCBI (National Center for Biotechnology Information) BLAST (Basic Local Alignment Search Tool) Tool (Version 2.2.29) was used to get the probes to the paired official circle RNAs with an identity equal to 100 as the matched criterion.Then, the official circle RNAs normalized signal intensity matrix was standardized using the Z score standardization formula [(*x* − *µ*)/*σ*, *x*: value, *µ*: average, *σ*: standard deviation] (16) performed using the Perl (Practical Extraction and Report Language, Version 5.30.2) software.

The DE circRNAs and miRNAs were identified using the limmapackage(version 3.30.0) of R (Version 3.5.1) software. DE circRNAs and miRNAs with |Fold change|(FC) > 1 and *p*-value < 0.05 were considered significant. For the DE circRNAs, we used the Venn software (http://bioinformatics.psb.ugent.be/webtools/Venn/) to obtain the common DE circRNAs in two independent cohorts.The DE mRNAs were identified using the DEseq package (Version 1.42.0) of R software with the same criteria as DE circRNAs.

### Prediction of circRNA-miRNA pairs

The circBase online website (http://www.circbase.org/) was used to obtain the basic data oncircRNAs (17). The target miRNAs and miRNA response elements(MREs) present in circRNAs were predicted using The Circular RNA Interactome (CircInteractome, https://circinteractome.nia.nih.gov/) (18). The predicted miRNAs were further selected as candidate target miRNAs for the DE circRNAs based on DE miRNAs of GSE97811.

### Survival analysis of De miRNAs

Kaplan-Meier plotter (http://kmplot.com/analysis/) analysis was performed to determine the survival-associated DE miRNAs. The BC database was used to estimate the prognostic values of thepredicted DE miRNAs. TheDE miRNA was divided into two groups based on median expression levels. *p* ≤ 0.05 was considered statistically significant.

### Prediction of miRNA-mRNA pairs

The miRNA-mRNA interactions were predicted using three online tools, including TargetScan(http://www.targetscan.org/) (19), miRDB (http://www.mirdb.org/) (20), and miRTarBase (http://mirtarbase.mbc.nctu.edu.tw/php/index.php) (21). The interactions predicted by all three websites were selectedas candidate targets to intersect with the DE mRNAs from the TCGA_BRCA dataset.

### Identification of prognostic predictors and construction of survival-related ceRNA networks

The survival-related DE mRNAs were selectedusing the same method as the DE miRNAs. Furthermore, a survival-related miRNA-miRNA-mRNA regulatory network was then constructed using a combination of circRNA-miRNA and miRNA-mRNA pairs. Cytoscape (http://cytoscape.org/; version 3.7.1) software was used for visualization (22). The top first degree, obtained using the Cytoscape cytoHubba plugin, was regarded as hub genes nodes.

### GO and KEGG enrichment analysis of survival-associated mRNAs

GO and KEGG annotations were downloaded from their official websites (http://current.geneontology.org/products/pages/downloads.html, https://www.genome.jp/kegg-bin/get_htext?hsa00001+3101, 2021.01.05download). The above original file combined with DE mRNAs were tidied into a 2 × 2 contingency table format using the Perl software, so that the survival-related DE mRNAs could be performed using the hypergeometric distribution formula of R software.We considered a *p*-value ≤ 0.05 and counted >2 as a statistically significant difference and significant enrichment, respectively.

### Hub gene GSEA

GSEA was performed to verify the prognosis of hub genes in BC and revealthe underlying pathway.The expression levels of genes fromthegenes chips were divided into two using the median. Moreover, the potential function of the hub genes was analyzed using the GSEA (version 4.1.0) (23).

### Breast cancer tissues

A total of 9 BC patients treated at the Department of Breast Surgery, The Affiliated Yantai Yuhuangding Hospital of Qingdao University were selected in our study. All patients signed informed consent forms approved by the Institutional Review Board of Yantai Yuhuangding Hospital (Certificate number: 2022-245) (23, 24).

### RNA isolation and Q-PCR

Total RNA was isolated using TRIzol reagent from the tissue samples of BC patients as per the manufacturer's direction (Shandong Sparkjade Biotechnology Co., Ltd.). After adding 0.5 µg RNA, each sample was reversed to cDNA by the SPARK script II RT Plus Kit (Shandong Sparkjade Biotechnology Co., Ltd.). Q-PCR analysis was performed using the SYBR Green qPCR Mix kit (With ROX) (Shandong Sparkjade Biotechnology Co., Ltd.), following the manufacturer's instructions. Finally, the expression levels of mRNA were calculated using the 2^–ΔΔCT^ formula.

### Statistical analysis

All statistical analyses were performed using R statistical software. Wilcoxon test was used to compare the differences between normal and tumor groups. A two-sided *p*-value < 0.05 indicated a statistically significant difference for all assays.

## Results

### Differential expression analysis

**Figure 2:**
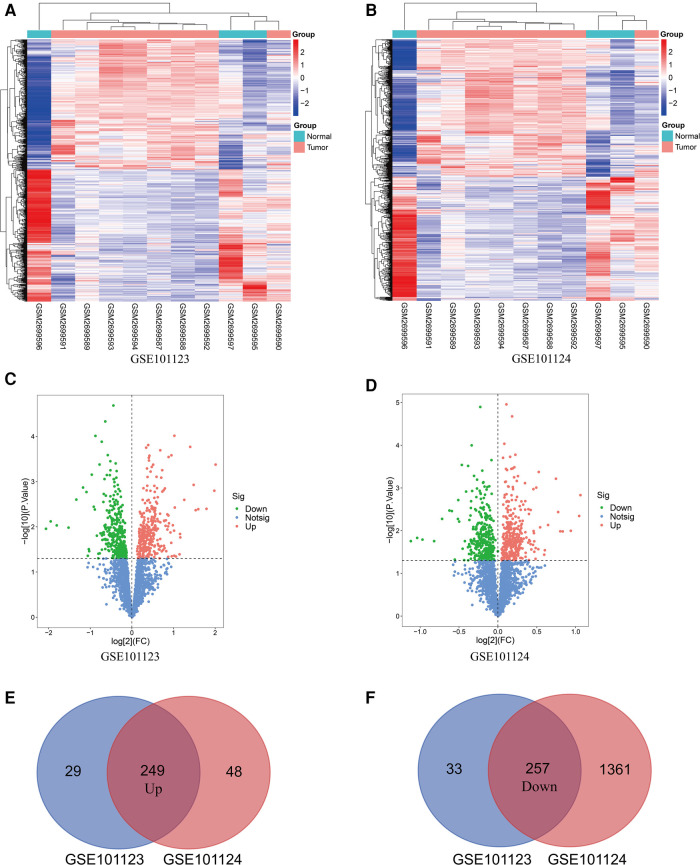
Differentially expressed circRNAs in BC patients compared with the Normal group. (**A,B**) Heatmap of the differentiallyexpressed circRNAs in BC based on GSE101123 and GSE101124. (**C,D**) Volcano map for all circRNAs in GSE101123 and GSE101124. (**E,F**) Overlap in DE circRNAs between GSE101123 and GSE101124.

**Figure 3:**
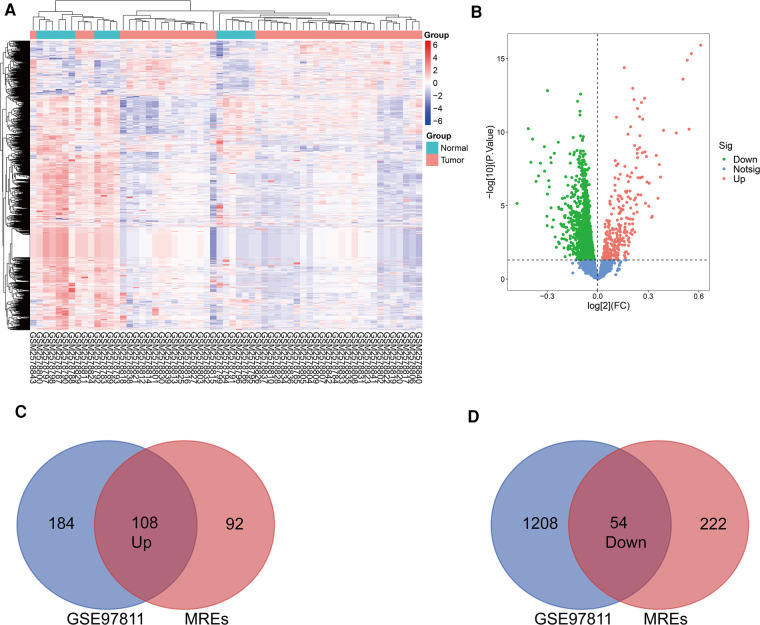
Identification of differentially expressed miRNAs. (**A**) Heatmap of the DE miRNAs from the GEOmicroarray in GSE97811. (**B**) Volcano map for all miRNAs in GSE97811. (**C,D**) Overlap between circRNA-related target miRNAs predicted bythe online tool and DEmiRNAs in GSE97811.

The basic information from three GEO datasets (GSE101123, GSE101123, and GSE97811) were used and are shown in Table 1. Each profile was analyzed using the limma package in R software. A |FC| > 1 and *p*-value < 0.05 were considered significant. Further, the DE circRNAs from the two circRNA data profiles were merged, and 249 up-regulated, and 257 down-regulated circRNAs were obtained (Figures 2E,F). The DE circRNAs were visualized using the heat map, volcano plot, and vein plot (Figures 2A–D). Simultaneously, 292 up-regulated and 1,262 down-regulated DE miRNAs were visualized using the heat map and volcano plot (Figures 3A,B). Furthermore, based on the initial top 10 candidates of DE circRNAs, circRNA–miRNA pair prediction was conducted using the online database CircInteractome. At last, the predicted miRNA response elements (MREs) of the circRNAs and DE miRNAs overlapped, and 108 up-regulated and 54 down-regulated target miRNAs based on the DE miRNAs were obtained using the Venn online tool (Figures 3C,D).

The complete TCGA_BRCA dataset of 113 normal and 1,102 cancer tissues were analyzed using the DEseq package in R software, and 3,696 up-regulated and 2,926 down-regulated DE mRNAs were identified using the same criterion as the above GEO dataset (Figure not shown).

### Survival analysis of the DE miRNAs

The survival analysis of the 108 up-regulated and 54 down-regulated DE miRNAs was studied using online Kaplan-Meier Plotter tools. Finally, the 22 up-regulated and 12 down-regulated DE miRNAs confirmed our expectations. Supplementary Figures S1, S2 depict the survival analyses of the miRNAs.

### Identification of prognostic predictors and construction of survival-related ceRNA networks

TargetScan (http://www.targetscan.org/), miRDB (http://www.mirdb.org/), and miRTarBase (http://mirtarbase.mbc.nctu.edu.tw/php/index.php) online tools were used to predict the potential interactions between miRNAs and mRNAs. Only target mRNAs recognized consistently through these three databases were selected as potential targets and intersected with DE mRNAs from the TCGA_BRCA dataset to identify candidate target mRNAs for the miRNAs. Finally, using Kaplan- Meier Plotter, 18 up-regulated and 28 down-regulated survival-related mRNAs (Supplementary Figures S3, S4) were selected.

**Figure 4:**
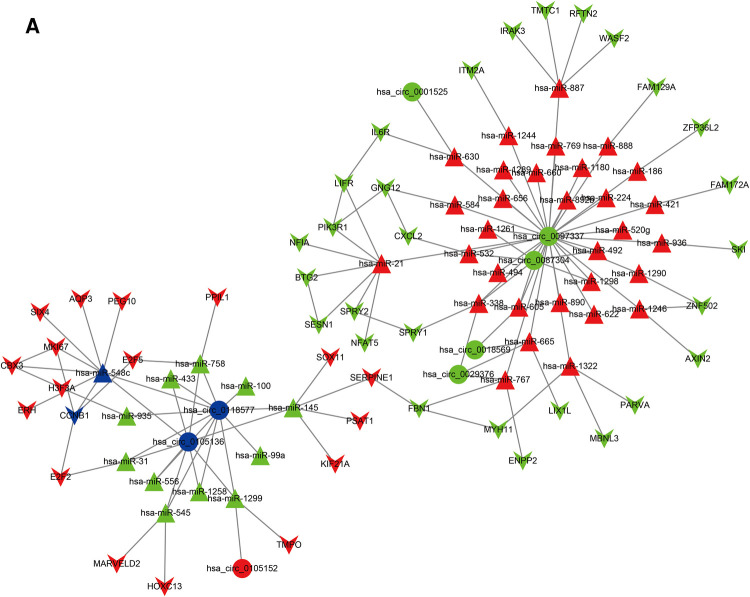
The ceRNA network of circRNA-miRNA-mRNA in BC. Circles represent circRNAs, triangles indicate miRNAs, and “V” indicates mRNAs. The red and green nodesindicate upregulation anddownregulation, respectively.Blue represents the hub nodes.

The BC survival-related circRNA-miRNA-mRNA ceRNA network was constructed using Cytoscape software (Figure 4A). The mRNAs from the ceRNA network were deposited into the STRING online database. Hub nodes of the mRNA network were identified using the Cytoscape cytoHubba plugin. Finally, two key circRNA-miRNA-mRNA axes (hsa_circ_0105136/hsa-miR-548c/CCNB1 and hsa_circ_0118577/hsa-miR-548c/CCNB1) were found.

### GO and KEGG enrichment analysis of survival-associated mRNAs

**Figure 5:**
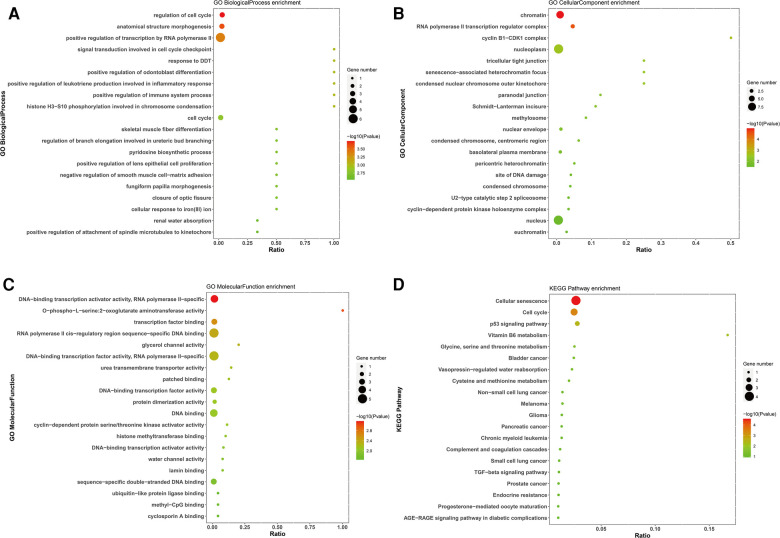
Go and KEGG enrichment analysis of up-regulated survival-associated mRNAs. (**A–C**) GO analysis of up-regulated mRNAs. (**D**) KEGG analysis of up-regulated mRNAs.

**Figure 6:**
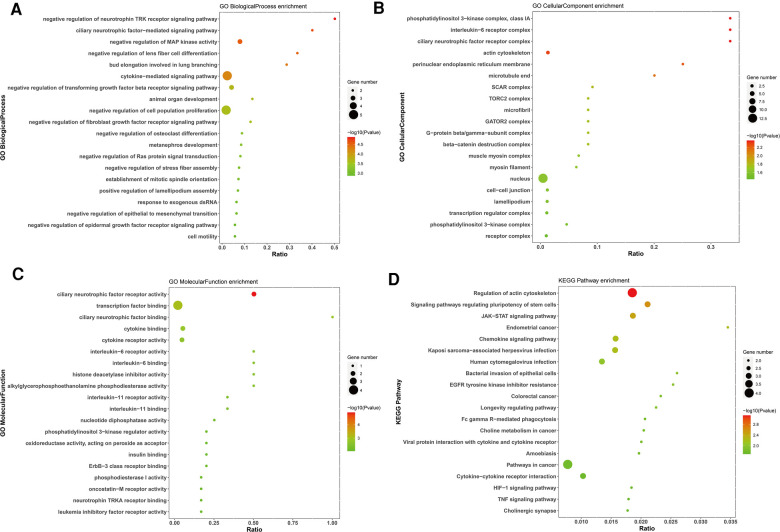
Go and KEGG enrichment analysis of down-regulated survival-associated mRNAs. (**A–C**) GO analysis of up-regulated mRNAs. (**D**) KEGG analysis of up-regulated mRNAs.

GO and KEGG enrichment analyses were performed to assess the biological functions and pathways of survival-associated DE mRNAs, which consisted of three different categories, namely, Biological Process (BP), Cell Component (CC), and Molecular Function (MF). The up-regulated survival-associated DE mRNAs were mostly involved in the “regulation of cell cycle” in the BP category, while the up-regulated survival-associated DE mRNAs were mostly involved in “chromatin”and “DNA-binding transcription activator activity, RNA polymerase II-specific” inthe CC and MF categories, respectively (Figures 5A–C). The up-regulated survival-associated DE mRNAs were most abundant in the “cellular senescence” KEGG pathway (Figure 5D). The down-regulated survival-associated DE mRNAs were most abundant in the “negative regulation of neurotrophin TRK receptor signaling pathway”,“interleukin-6 receptor complex”, and “ciliary neurotrophic factor receptor activity” (Figures 6A–C). Moreover, the down-regulated survival-associated DE mRNAs were mostly associated with the “regulation of actin cytoskeleton” KEGG pathway (Figure 6D).

### Hub gene GSEA

**Figure 7:**
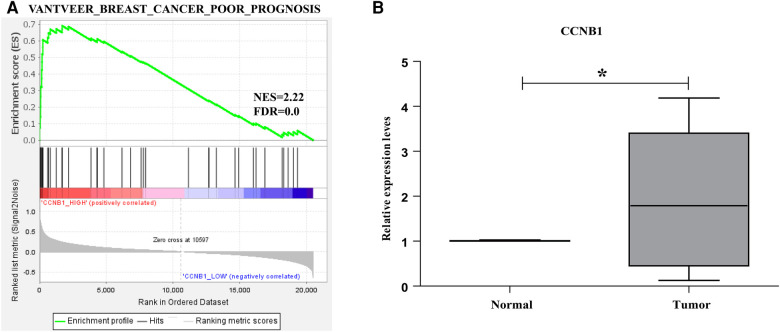
Enrichment plots from GSEA. (**A**) CCNB1 was positively related tothe poor prognosis of BC. (**B**) *CCNB1* genes validation in nine BC patient's tissues.

GSEA was performed to reveal the underlying pathway from the GSEA dataset to validate the prognosis of hub genes in BC. The expression levels of these genes from the TCGA_BRCA were divided into two parts using the median. The “VANTVEER_BREAST_CANCER_POOR_PROGNOSIS” geneset was analyzed. The findings suggested that CCNB1 was positively correlated with the poor prognosis in BC (Figure 7).

### Validation of the hub genes using BC samples

The mRNA from nine BC patients' carcinomas and nearby normal tissues was extracted and reverse-transcribed to cDNA. Following that, the expression level of hub genes (*CCNB1*) was investigated using Q-PCR.The results revealed that the *CCNB1* was highly expressed in the carcinoma tissues (Figure 7B), consistent with our results.

## Discussion

Although the majority of the genome is transcribed, only about 1%–2% of it encodes proteins, and the rest of the genome may express non-coding RNAs (25). The significance of non-coding RNAs in tumor progression has been the main research focus in recent years. However, the biological and molecular functions of non-coding RNAs in various cancers remain unknown. BC is the leading cause of cancer-related death in women (26), and despite advances in treatment, the death rate remains high (27).

Here, we combined the TCGA_BRCA mRNA dataset with circRNA and miRNA expression profiles of BC tissues from the GEO database to demonstrate the possible mechanisms of BC. A total of 22 up-regulated and 12 down-regulated survival-related DE miRNAs were identified. Furthermore, target mRNAs were predicted using three online tools, and overlapping survival-related 18 up-regulated and 28 down-regulated DE mRNAs were identified. Moreover, the DE mRNAs were consistent with the TCGA_BRCA mRNA dataset. The GO and KEGG enrichment analyses of potential DE mRNAs were carried out using the hypergeometry of R software. The up-regulated DE mRNAs were mostly involved in the “structural constituent of cytoskeleton” molecular function and the “Salmonella infection” KEGG pathway. The down-regulated DE mRNAs were mostly enriched in “signaling adaptor activity” molecular functions and “pathways in cancer” KEGG pathways. Finally, a ceRNA network was constructed. From the ceRNA network and mRNA network, we found that the hsa_circ_0105136/hsa-miR-548c/CCNB1 and hsa_circ_0118577/hsa-miR-548c/CCNB1axes were the most significant. In addition, these axes were found to be correlated with the poorprognosis of BC.

Hsa_circ_0105136/hsa-miR-548c/CCNB1 is an indispensable axis in the ceRNA network, suggesting that it could be a crucial mechanism leading to BC. CCNB is highly expressed in various cancers, including colorectal cancer (28), bladder cancer (29), BC (30), and pituitary cancer (31). Furthermore, it participates in cell proliferation, invasion, and metastasis (32). It may serve as a biomarker forestrogen receptor-positive BC (33). Simultaneously, it can activate the epithelial-mesenchymal transition (EMT) process (32). Our findings may provide new insight into the biological mechanism of CCNB1 in the development and progression of BC. Aberrant miRNAs have been reported to play an important role in the progression of cancers, including BC (32); for example, miR-125b is down-regulated in BC and inhibits cell proliferation, invasion, and migration (34, 35). miR-548 could potentially prevent HIV, HCV, and HBV infection (36). However, the role of miR-548c in the breast hasnot been elucidated. miR-548c belongs tothe miR-548 family, and our findings revealed that it was down-regulated in BC, suggesting that high expression of miR-548c inhibits BC cell proliferation and invasion. However, the relationship between hsa-miR-548c and CCNB1 remains unknown. In summary, we hypothesized that hsa_circ_0105136/hsa_circ_0118577 influenced BC progression *via* the miR-548c/CCNB1 axis.

## Conclusions

We constructed an underlying ceRNA network based on the above findings, which provides new insight intothe potential mechanisms of BC and provides a candidate biomarker for BC. The hsa_circ_0105136/hsa-miR-548c/CCNB1 and hsa_circ_0118577/hsa-miR-548c/CCNB1 axes, in particular, may be key pathways impacting BC progression and prognosis. This analysis contributes to identifying novel diagnostic biomarkers and therapeutic targets for BC patients.

## Data Availability

Publicly available datasets were analyzed in this study. This data can be found here: NCBI: GSE101123, GSE97811 and GSE101124.

## Supplementary material

Supplement 1 Up-regulated DE miRNAs survival lines.

Supplement 2 Down-regulated DE miRNAs survival lines.

Supplement 3 Up-regulated DE mRNAs survival lines.

Supplement 4 Down-regulated DE mRNAs survival lines.
