# Supplementary figures and images for: Construction and analysis of a survival-associated competing endogenous RNA network in breast cancer
Source: Front Surg. 2023 Jan 6;9:1021195. doi: 10.3389/fsurg.2022.1021195 (PMC9852745; doi:10.3389/fsurg.2022.1021195)

### Supplement 1. Upregulated DE miRNAs survival lines.

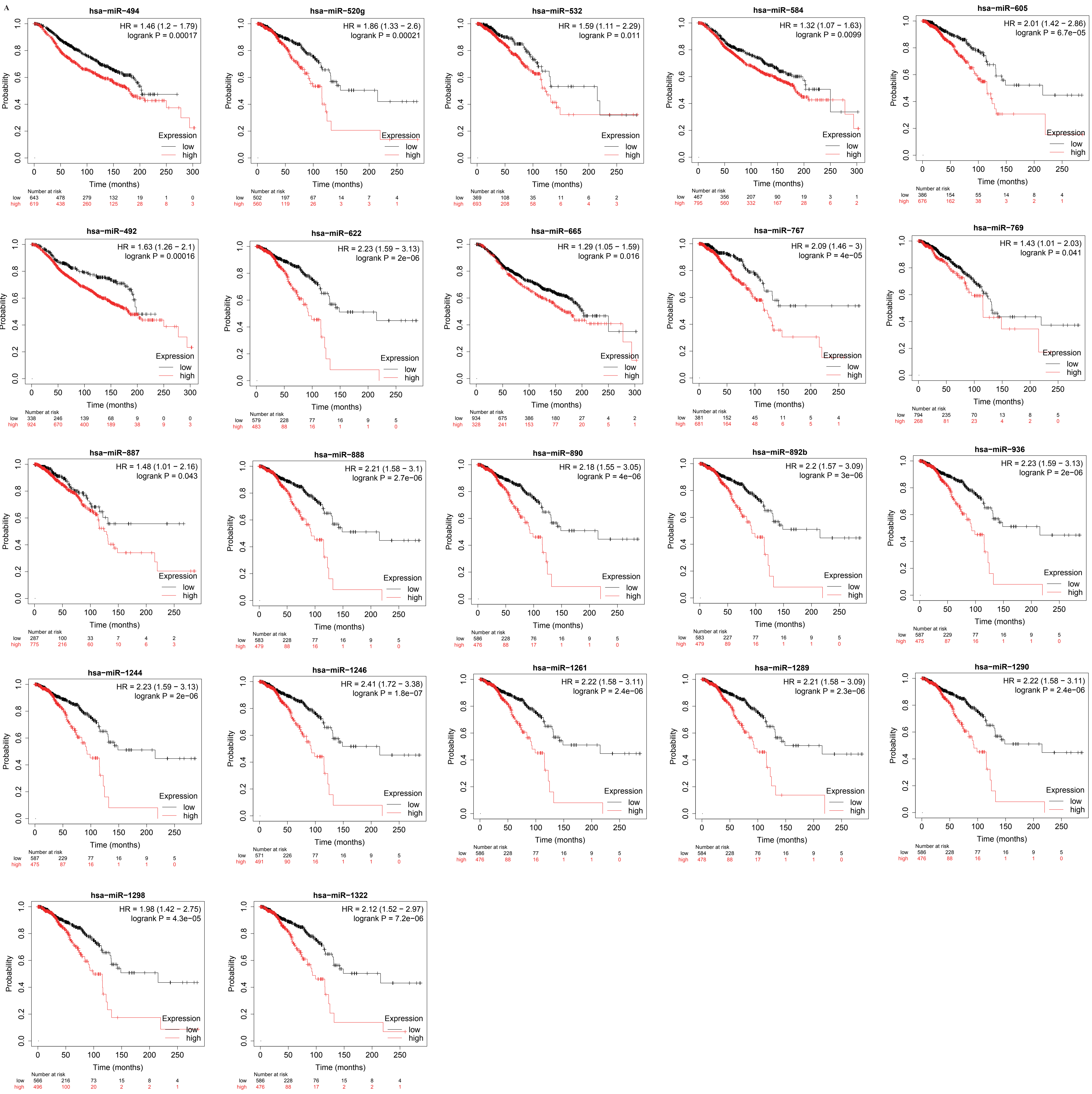

Supplement: Supplement 1 — Up-regulated DE miRNAs survival lines. [file Datasheet1.pdf]

Supplement 2. Downregulated DE miRNAs survival lines.

A

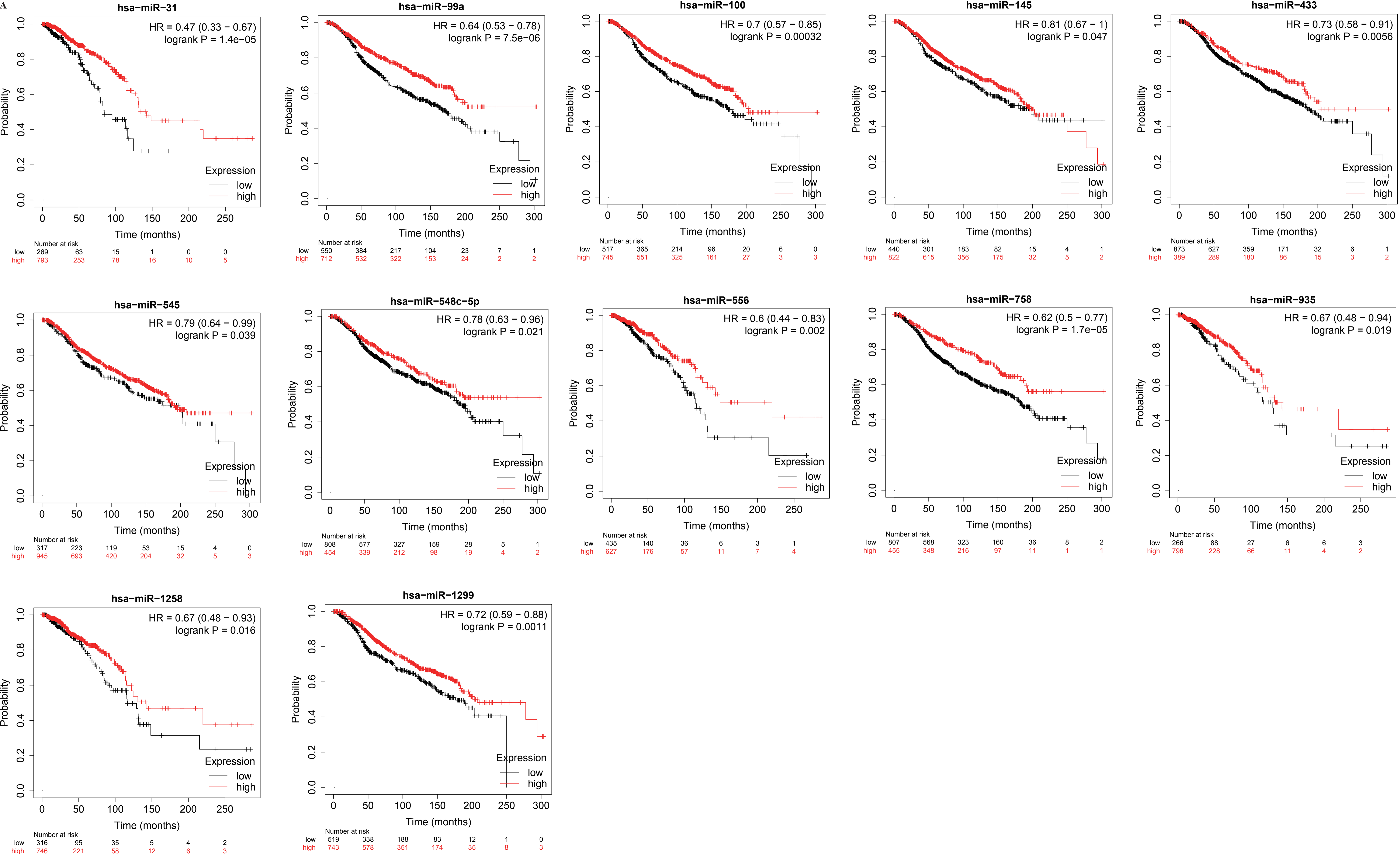

Supplement: Supplement 2 — Down-regulated DE miRNAs survival lines. [file Datasheet2.pdf]

Supplement 3. Upregulated DE mRNAs survival lines.

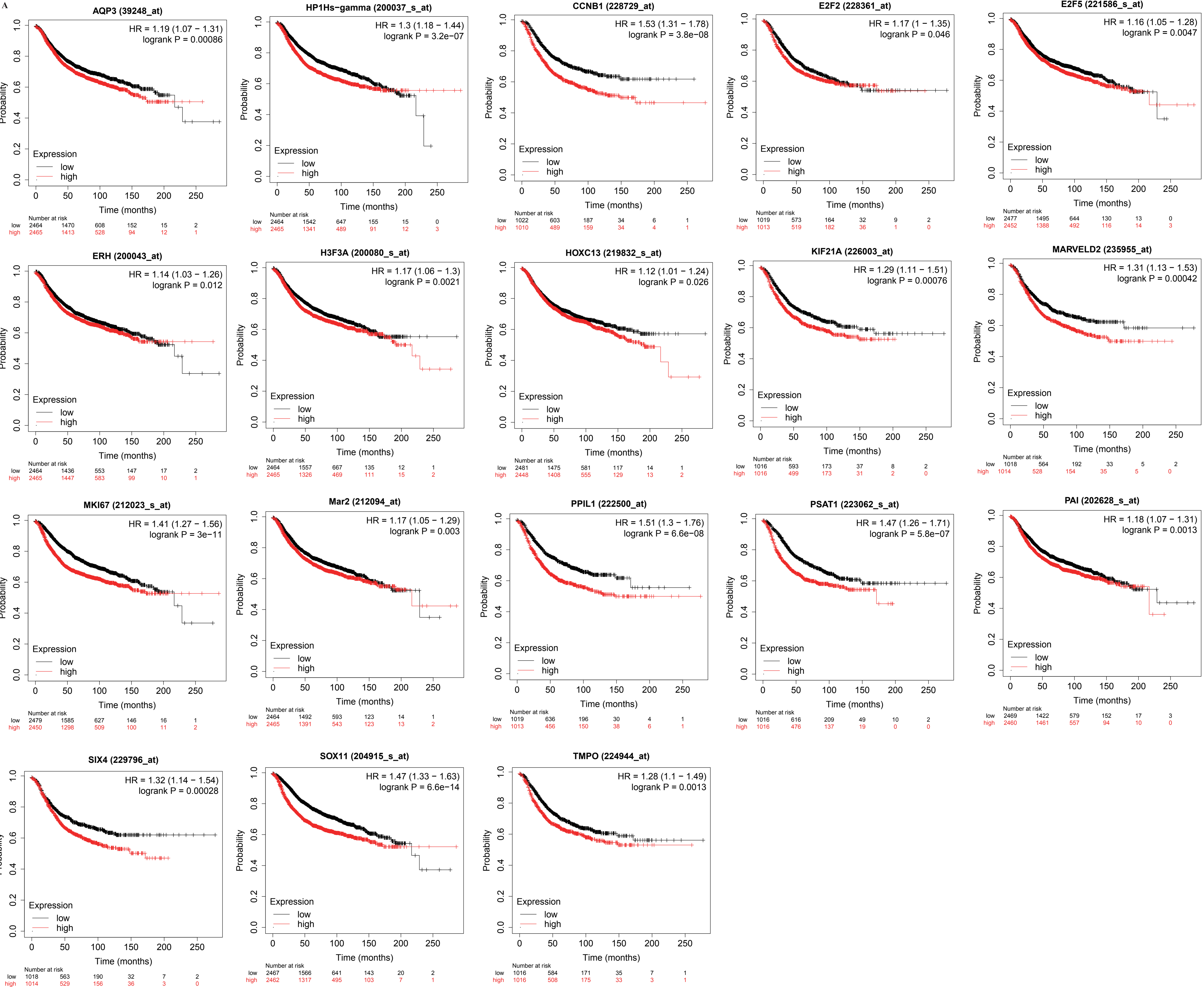

Supplement: Supplement 3 — Up-regulated DE mRNAs survival lines. [file Datasheet3.pdf]

Supplement 4. Downregulated DE mRNAs survival lines.

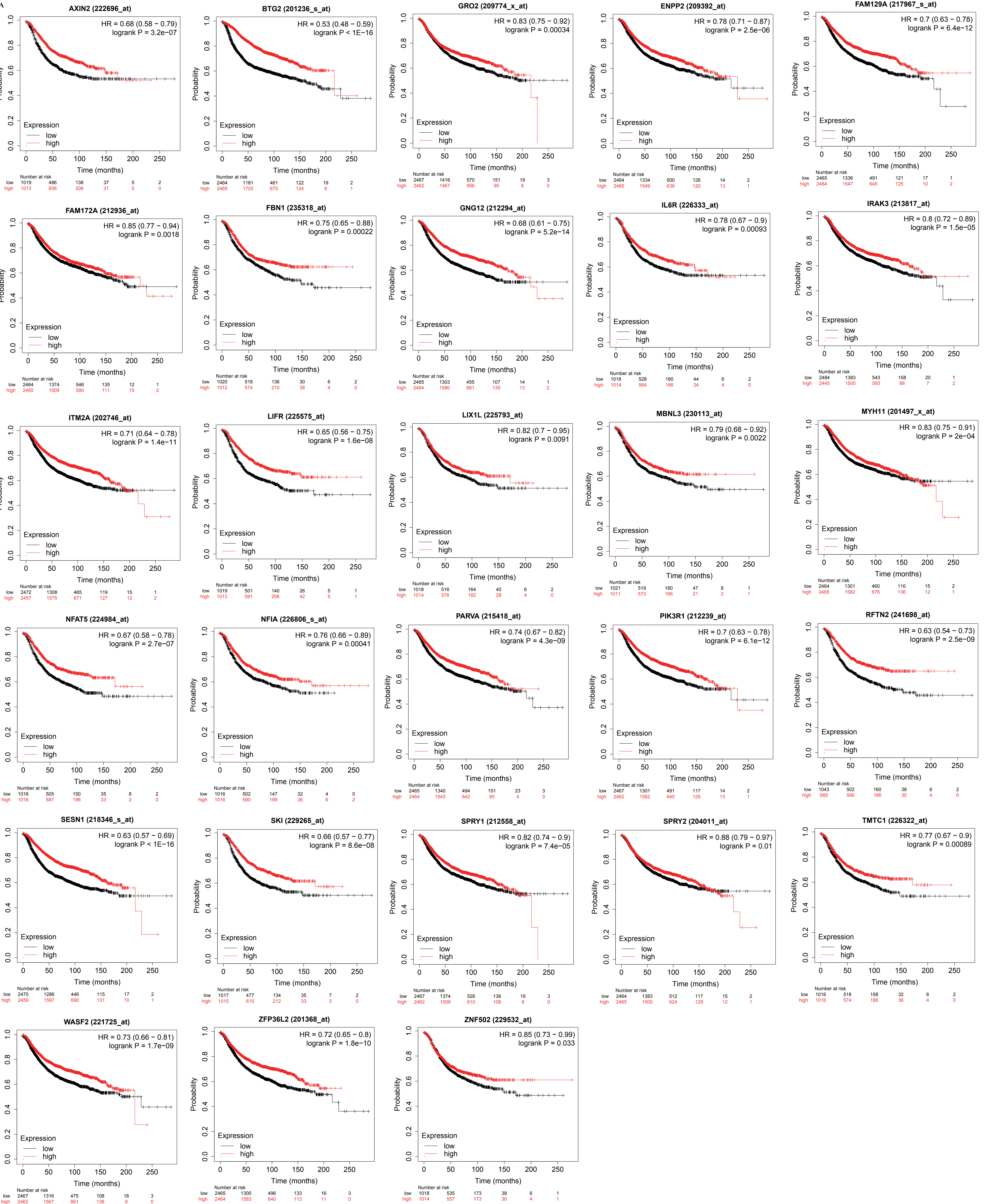

Supplement: Supplement 4 — Down-regulated DE mRNAs survival lines. [file Datasheet4.pdf]

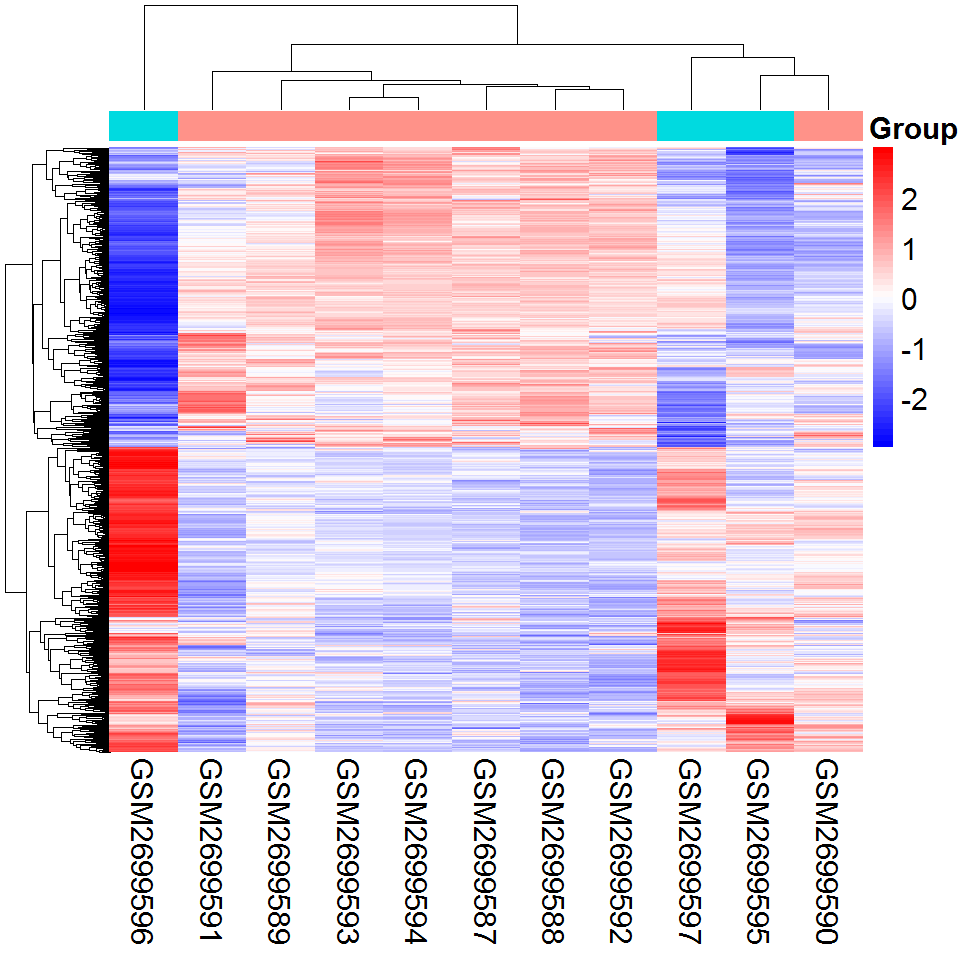

Supplement: Supplementary file 5 [file Datasheet5.zip › Figure_2/GSE101123.Sig.Tumor.Normal.heatmap.png]

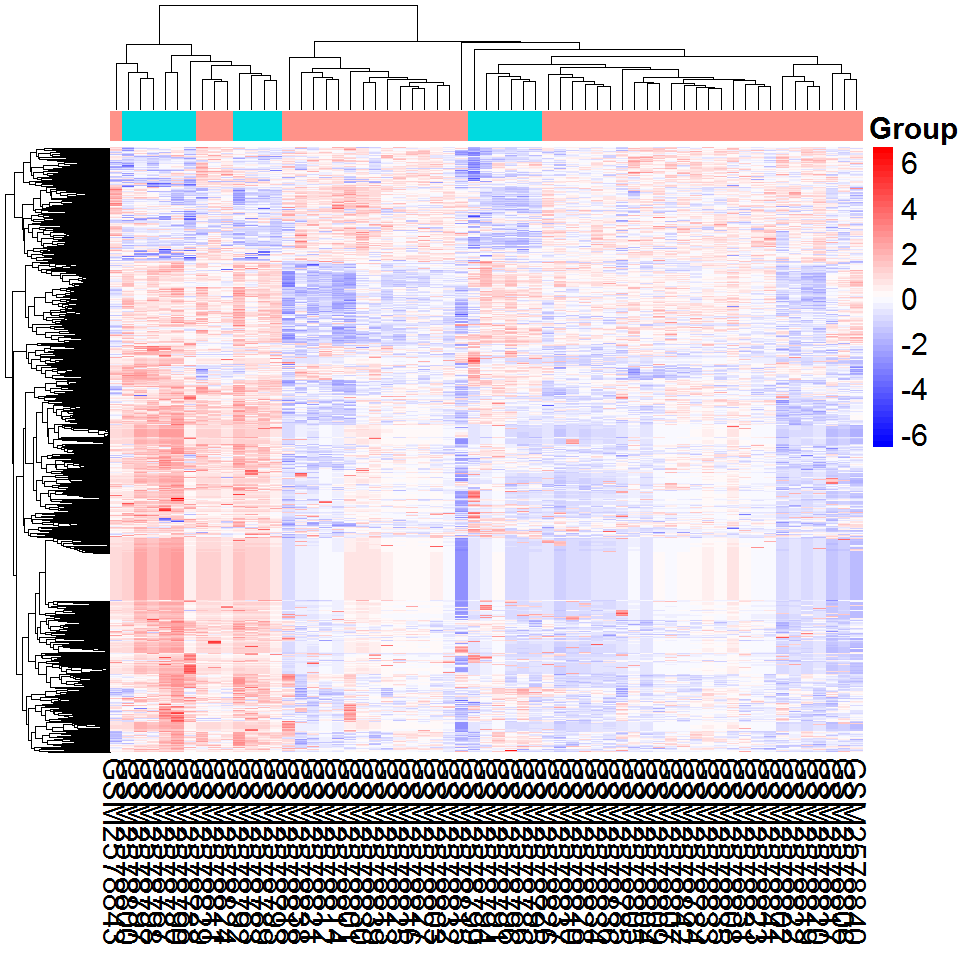

Supplement: Supplementary file 6 [file Datasheet6.zip › Figure_3/GSE97811.Sig.Tumor.Normal.heatmap.png]

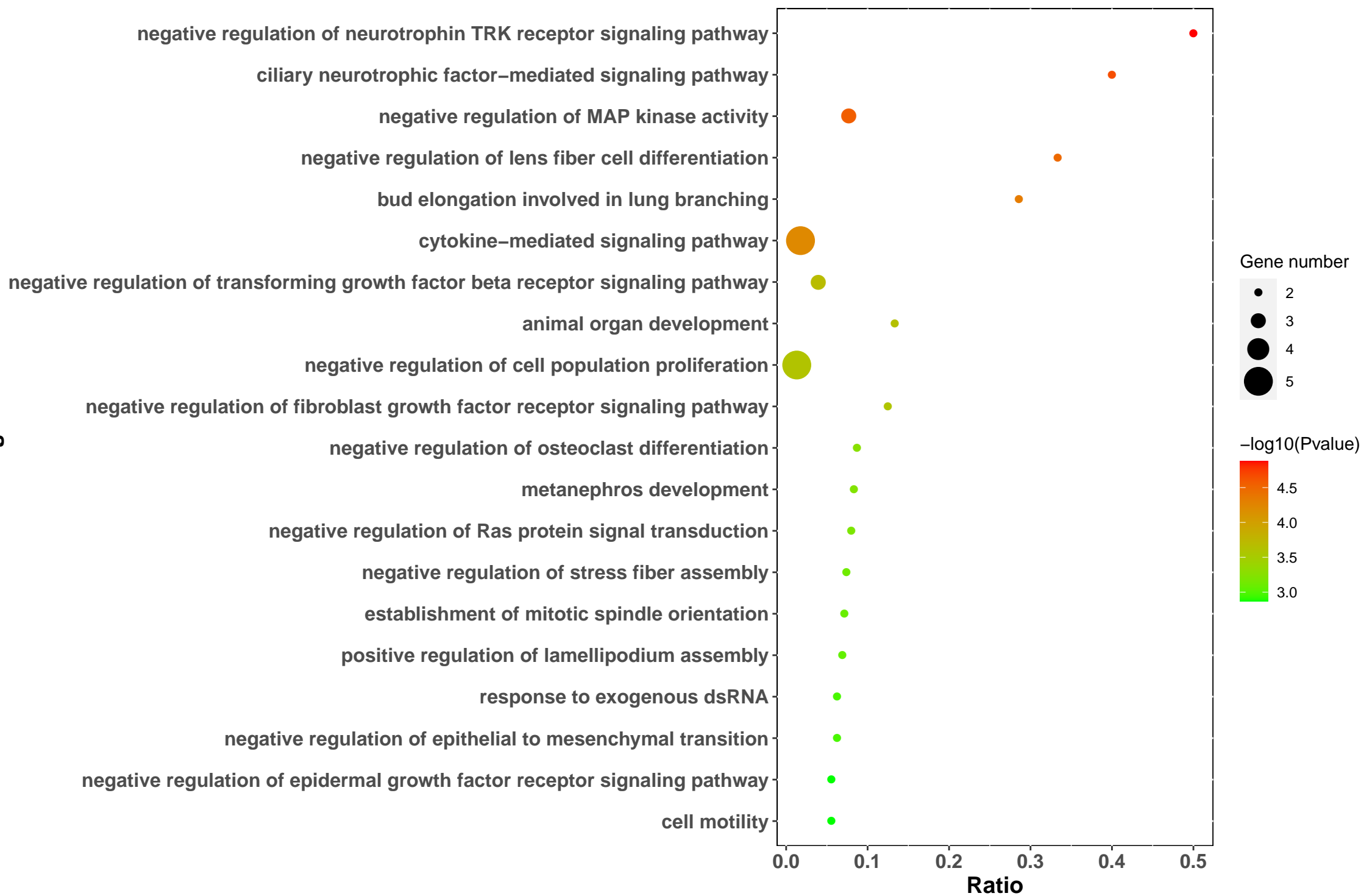

Supplement: Supplementary file 9 [file Datasheet9.zip › Figure_6/GO.BP.(Sample).enrichment.pdf]

GO CellularComponent enrichment

GO CellularComponent

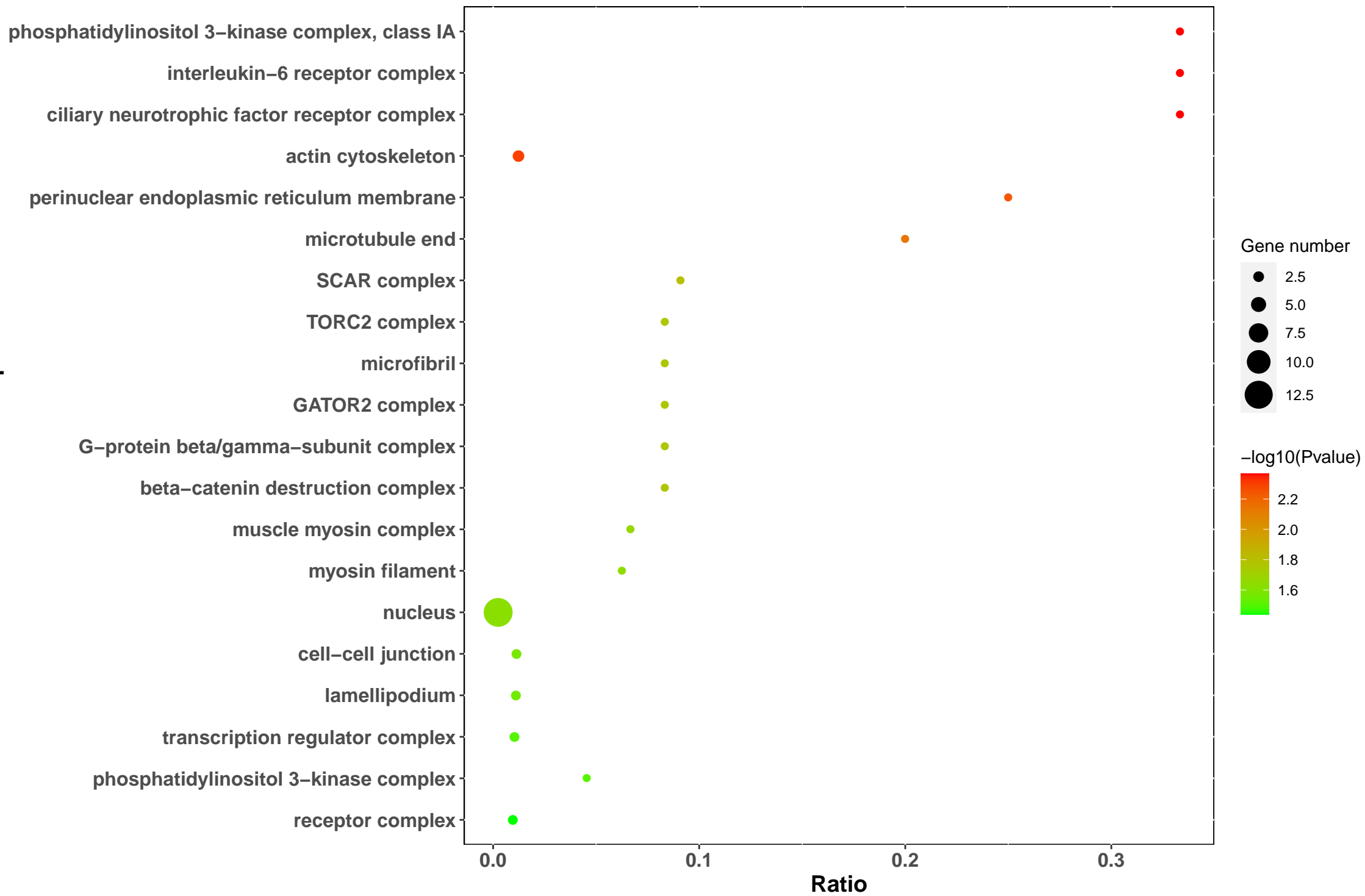

Supplement: Supplementary file 9 [file Datasheet9.zip › Figure_6/GO.CC.(Sample).enrichment.pdf]

## GO MolecularFunction enrichment

GO MolecularFunction

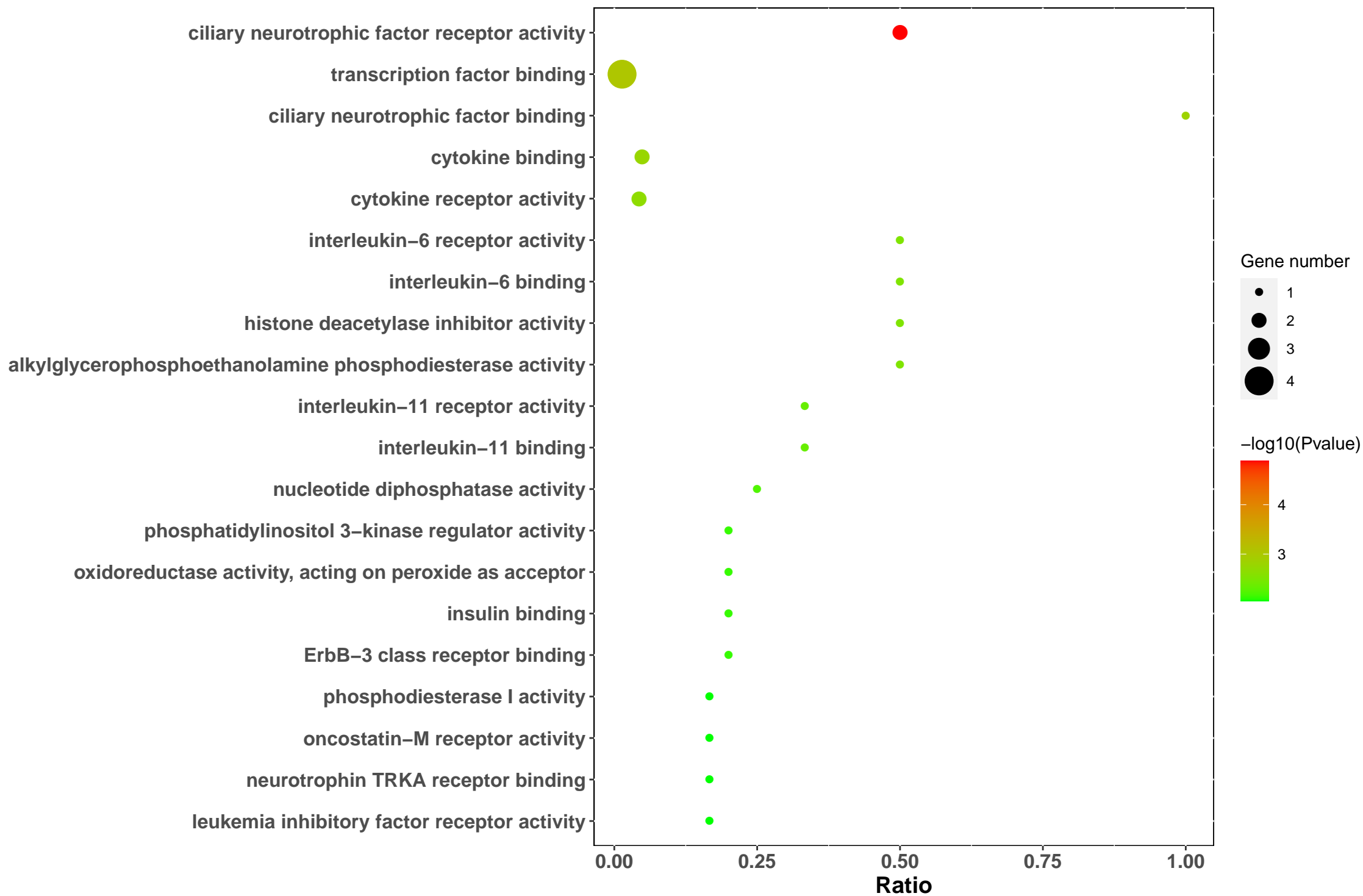

Supplement: Supplementary file 9 [file Datasheet9.zip › Figure_6/GO.MF.(Sample).enrichment.pdf]

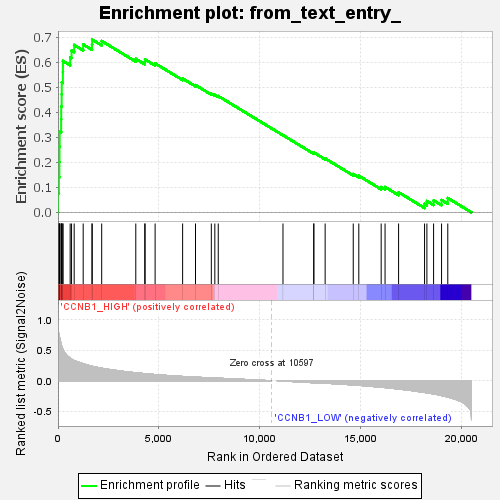

Supplement: Supplementary file 10 [file Datasheet10.zip › Figure_7/GSEA-CCNB1_VANTVEER_BREAST_CANCER_POOR_PROGNOSIS.Gsea.1629329727900/enplot_from_text_entry__3.png]

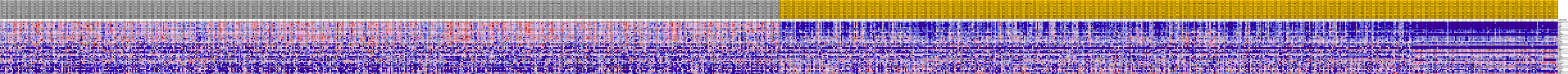

Supplement: Supplementary file 10 [file Datasheet10.zip › Figure_7/GSEA-CCNB1_VANTVEER_BREAST_CANCER_POOR_PROGNOSIS.Gsea.1629329727900/from_text_entry__4.png]

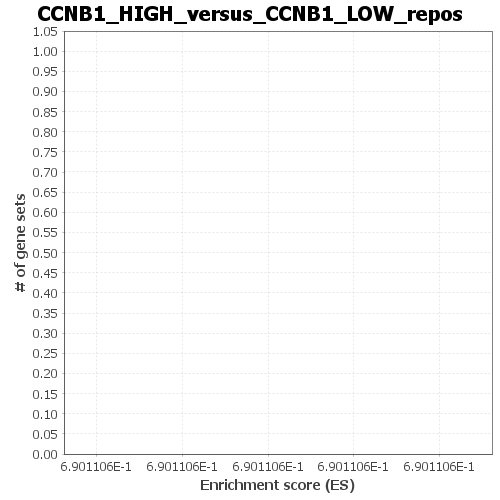

Supplement: Supplementary file 10 [file Datasheet10.zip › Figure_7/GSEA-CCNB1_VANTVEER_BREAST_CANCER_POOR_PROGNOSIS.Gsea.1629329727900/global_es_histogram.png]

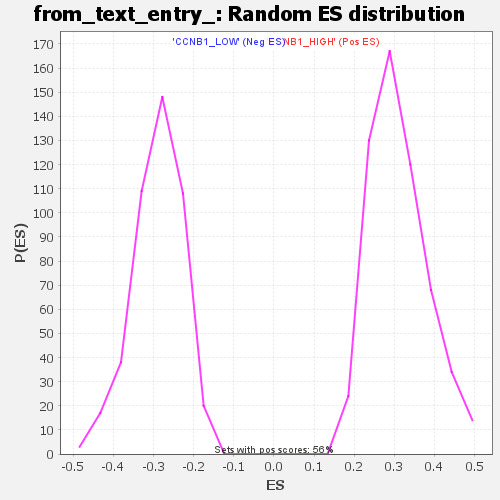

Supplement: Supplementary file 10 [file Datasheet10.zip › Figure_7/GSEA-CCNB1_VANTVEER_BREAST_CANCER_POOR_PROGNOSIS.Gsea.1629329727900/gset_rnd_es_dist_5.png]

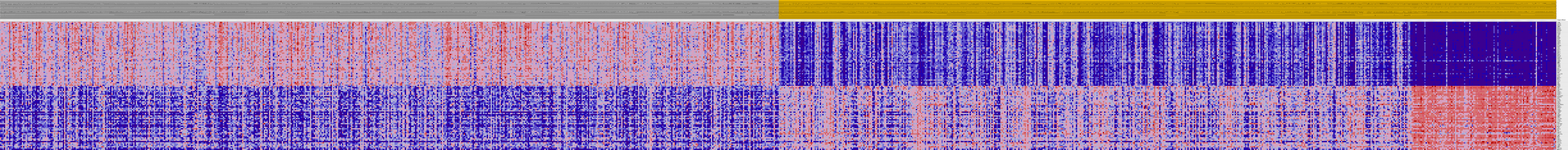

Supplement: Supplementary file 10 [file Datasheet10.zip › Figure_7/GSEA-CCNB1_VANTVEER_BREAST_CANCER_POOR_PROGNOSIS.Gsea.1629329727900/heat_map_1.png]

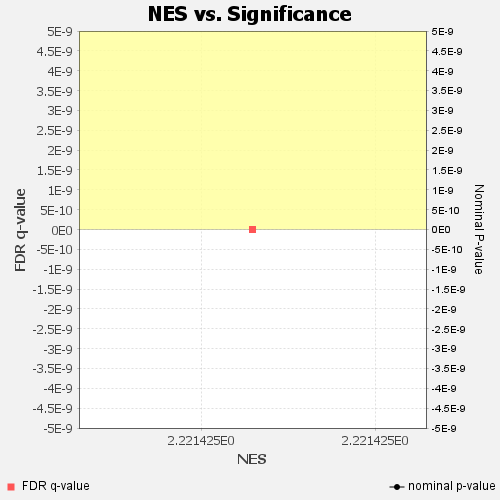

Supplement: Supplementary file 10 [file Datasheet10.zip › Figure_7/GSEA-CCNB1_VANTVEER_BREAST_CANCER_POOR_PROGNOSIS.Gsea.1629329727900/pvalues_vs_nes_plot.png]

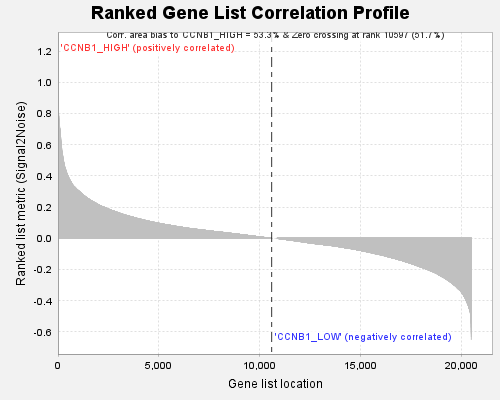

Supplement: Supplementary file 10 [file Datasheet10.zip › Figure_7/GSEA-CCNB1_VANTVEER_BREAST_CANCER_POOR_PROGNOSIS.Gsea.1629329727900/ranked_list_corr_2.png]
