# Supplementary material for: Construction and analysis of a survival-associated competing endogenous RNA network in breast cancer
Source: Front Surg. 2023 Jan 6;9:1021195. doi: 10.3389/fsurg.2022.1021195 (PMC9852745; doi:10.3389/fsurg.2022.1021195)
Supplement: Supplementary file 10 [file Datasheet10.zip › Figure_7/GSEA-CCNB1_VANTVEER_BREAST_CANCER_POOR_PROGNOSIS.Gsea.1629329727900/from_text_entry_.html]

Details for gene set from\_text\_entry\_[GSEA]

|  || Dataset | tumor\_normal\_normal.CCNB1\_LOW\_vs\_CCNB1\_HIGH.cls #CCNB1\_HIGH\_versus\_CCNB1\_LOW.CCNB1\_LOW\_vs\_CCNB1\_HIGH.cls #CCNB1\_HIGH\_versus\_CCNB1\_LOW\_repos |
| Phenotype | CCNB1\_LOW\_vs\_CCNB1\_HIGH.cls#CCNB1\_HIGH\_versus\_CCNB1\_LOW\_repos |
| Upregulated in class | CCNB1\_HIGH |
| GeneSet | from\_text\_entry\_ |
| Enrichment Score (ES) | 0.69011056 |
| Normalized Enrichment Score (NES) | 2.221425 |
| Nominal p-value | 0.0 |
| FDR q-value | 0.0 |
| FWER p-Value | 0.0 |
Table: GSEA Results Summary

  

Fig 1: Enrichment plot: from\_text\_entry\_      
 Profile of the Running ES Score & Positions of GeneSet Members on the Rank Ordered List

  

| SYMBOL | TITLE | RANK IN GENE LIST | RANK METRIC SCORE | RUNNING ES | CORE ENRICHMENT || 1 | NUSAP1 | na | 5 | 0.889 | 0.0764 | Yes |
| 2 | MELK | na | 44 | 0.762 | 0.1402 | Yes |
| 3 | NDC80 | na | 70 | 0.722 | 0.2012 | Yes |
| 4 | CENPA | na | 76 | 0.717 | 0.2628 | Yes |
| 5 | PRC1 | na | 84 | 0.704 | 0.3231 | Yes |
| 6 | CCNE2 | na | 156 | 0.603 | 0.3716 | Yes |
| 7 | MCM6 | na | 160 | 0.601 | 0.4233 | Yes |
| 8 | RFC4 | na | 181 | 0.571 | 0.4715 | Yes |
| 9 | GMPS | na | 186 | 0.560 | 0.5196 | Yes |
| 10 | DIAPH3 | na | 241 | 0.512 | 0.5611 | Yes |
| 11 | DTL | na | 244 | 0.511 | 0.6051 | Yes |
| 12 | UCHL5 | na | 603 | 0.369 | 0.6194 | Yes |
| 13 | MTDH | na | 672 | 0.354 | 0.6466 | Yes |
| 14 | TMEM65 | na | 802 | 0.330 | 0.6687 | Yes |
| 15 | DCK | na | 1249 | 0.278 | 0.6709 | Yes |
| 16 | PITRM1 | na | 1686 | 0.238 | 0.6701 | Yes |
| 17 | LPCAT1 | na | 1695 | 0.237 | 0.6901 | Yes |
| 18 | TSPYL5 | na | 2167 | 0.205 | 0.6847 | No |
| 19 | EXT1 | na | 3858 | 0.130 | 0.6134 | No |
| 20 | ESM1 | na | 4303 | 0.115 | 0.6016 | No |
| 21 | NMU | na | 4320 | 0.115 | 0.6107 | No |
| 22 | SERF1A | na | 4816 | 0.101 | 0.5952 | No |
| 23 | GNAZ | na | 6181 | 0.070 | 0.5346 | No |
| 24 | RAB6B | na | 6819 | 0.058 | 0.5084 | No |
| 25 | MMP9 | na | 7604 | 0.046 | 0.4741 | No |
| 26 | AP2B1 | na | 7778 | 0.043 | 0.4694 | No |
| 27 | OXCT1 | na | 7948 | 0.041 | 0.4647 | No |
| 28 | IGFBP5 | na | 11158 | -0.004 | 0.3082 | No |
| 29 | COL4A2 | na | 12684 | -0.032 | 0.2364 | No |
| 30 | EBF4 | na | 12689 | -0.032 | 0.2390 | No |
| 31 | BBC3 | na | 13249 | -0.041 | 0.2152 | No |
| 32 | CDC42BPA | na | 14643 | -0.068 | 0.1530 | No |
| 33 | ALDH4A1 | na | 14919 | -0.075 | 0.1459 | No |
| 34 | MS4A7 | na | 16028 | -0.106 | 0.1009 | No |
| 35 | GSTM3 | na | 16221 | -0.112 | 0.1011 | No |
| 36 | SLC2A3 | na | 16892 | -0.136 | 0.0801 | No |
| 37 | STK32B | na | 18180 | -0.192 | 0.0337 | No |
| 38 | FGF18 | na | 18296 | -0.197 | 0.0451 | No |
| 39 | SCUBE2 | na | 18628 | -0.215 | 0.0475 | No |
| 40 | TGFB3 | na | 19023 | -0.243 | 0.0492 | No |
| 41 | FLT1 | na | 19332 | -0.267 | 0.0571 | No |
Table: GSEA details [plain text format]

  

Fig 2: from\_text\_entry\_      
 Blue-Pink O' Gram in the Space of the Analyzed GeneSet

  

Fig 3: from\_text\_entry\_: Random ES distribution      
 Gene set null distribution of ES for **from\_text\_entry\_**

  
