# Supplementary material for: Construction and analysis of a survival-associated competing endogenous RNA network in breast cancer
Source: Front Surg. 2023 Jan 6;9:1021195. doi: 10.3389/fsurg.2022.1021195 (PMC9852745; doi:10.3389/fsurg.2022.1021195)
Supplement: Supplementary file 10 [file Datasheet10.zip › Figure_7/GSEA-CCNB1_VANTVEER_BREAST_CANCER_POOR_PROGNOSIS.Gsea.1629329727900/gsea_report_for_CCNB1_LOW_1629329727900.html]

Report for CCNB1\_LOW 1629329727900 [GSEA]

| GS  follow link to MSigDB | GS DETAILS | SIZE | ES | NES | NOM p-val | FDR q-val | FWER p-val | RANK AT MAX | LEADING EDGE |Table: Gene sets enriched in phenotype **CCNB1\_LOW (607 samples)**[plain text format]****

  
