# Supplementary material for: Construction and analysis of a survival-associated competing endogenous RNA network in breast cancer
Source: Front Surg. 2023 Jan 6;9:1021195. doi: 10.3389/fsurg.2022.1021195 (PMC9852745; doi:10.3389/fsurg.2022.1021195)
Supplement: Supplementary file 10 [file Datasheet10.zip › Figure_7/GSEA-CCNB1_VANTVEER_BREAST_CANCER_POOR_PROGNOSIS.Gsea.1629329727900/heat_map_corr_plot.html]

Heat map and correlation plot for tumor\_normal\_normal.CCNB1\_LOW\_vs\_CCNB1\_HIGH.cls#CCNB1\_HIGH\_versus\_CCNB1\_LOW  

Fig 1: heat\_map      
 Heat Map of the top 50 features for each phenotype in tumor\_normal\_normal.CCNB1\_LOW\_vs\_CCNB1\_HIGH.cls#CCNB1\_HIGH\_versus\_CCNB1\_LOW

  
  

Fig 2: Ranked Gene List Correlation Profile      
 Ranked list correlations for tumor\_normal\_normal.CCNB1\_LOW\_vs\_CCNB1\_HIGH.cls#CCNB1\_HIGH\_versus\_CCNB1\_LOW

  
  
    
