# Supplementary material for: Construction and analysis of a survival-associated competing endogenous RNA network in breast cancer
Source: Front Surg. 2023 Jan 6;9:1021195. doi: 10.3389/fsurg.2022.1021195 (PMC9852745; doi:10.3389/fsurg.2022.1021195)
Supplement: Supplementary file 10 [file Datasheet10.zip › Figure_7/GSEA-CCNB1_VANTVEER_BREAST_CANCER_POOR_PROGNOSIS.Gsea.1629329727900/index.html]

Index for xtools.gsea.Gsea CCNB1\_VANTVEER\_BREAST\_CANCER\_POOR\_PROGNOSIS.Gsea.1629329727900

### GSEA Report for Dataset tumor\_normal

#### Enrichment in phenotype: **CCNB1\_HIGH (608 samples)**

- 1 / 1 gene sets are upregulated in phenotype **CCNB1\_HIGH**- 1 gene sets are significant at FDR < 25%- 1 gene sets are significantly enriched at nominal pvalue < 1%- 1 gene sets are significantly enriched at nominal pvalue < 5%- Snapshot of enrichment results- Detailed enrichment results in html format- Detailed enrichment results in TSV format (tab delimited text)- Guide to interpret results

#### Enrichment in phenotype: **CCNB1\_LOW (607 samples)**

- None of the gene sets are enriched in phenotype **CCNB1\_LOW**- Guide to interpret results

#### Dataset details

- The dataset has 20501 features (genes)- No probe set => gene symbol collapsing was requested, so all 20501 features were used

#### Gene set details

- Gene set size filters (min=15, max=500) resulted in filtering out 0 / 1 gene sets- The remaining 1 gene sets were used in the analysis- List of gene sets used and their sizes (restricted to features in the specified dataset)

#### Gene markers for the **CCNB1\_HIGH** *versus* **CCNB1\_LOW** comparison

- The dataset has 20501 features (genes)- # of markers for phenotype **CCNB1\_HIGH**: 10597 (51.7% ) with correlation area 53.3%- # of markers for phenotype **CCNB1\_LOW**: 9904 (48.3% ) with correlation area 46.7%- Detailed rank ordered gene list for all features in the dataset- Heat map and gene list correlation  profile for all features in the dataset

#### Global statistics and plots

- Plot of p-values *vs.* NES- Global ES histogram

#### Other

- Parameters used for this analysis

#### Comments

- There were duplicate row identifiers in the specified dataset. One id was arbitarilly choosen. Details are below
  Generally, this is OK, but if you want to avoid this automagic, edit your dataset so that all row ids are unique
  # of row ids in original dataset: 20502
  # of row UNIQUE ids in original dataset: 20501
  # The duplicates were
  SLC35E2- Timestamp used as random seed: 1629329729572

---

Report: CCNB1\_VANTVEER\_BREAST\_CANCER\_POOR\_PROGNOSIS.Gsea.1629329727900.rpt   by user: Administrator

xtools.gsea.Gsea [Thu, Aug 19, '21 7 AM 35]

Website: www.gsea-msigdb.org/gsea
Questions & Suggestions: Contact page
